# The Omega*-*3 Fatty Acid Eicosapentaenoic Acid Is Required for Normal Alcohol Response Behaviors in *C. elegans*

**DOI:** 10.1371/journal.pone.0105999

**Published:** 2014-08-27

**Authors:** Richard C. Raabe, Laura D. Mathies, Andrew G. Davies, Jill C. Bettinger

**Affiliations:** 1 Department of Pharmacology and Toxicology, Virginia Commonwealth University, Richmond, Virginia, United States of America; 2 VCU-Alcohol Research Center, Virginia Commonwealth University, Richmond, Virginia, United States of America; Max Delbrueck Center for Molecular Medicine, Germany

## Abstract

Alcohol addiction is a widespread societal problem, for which there are few treatments. There are significant genetic and environmental influences on abuse liability, and understanding these factors will be important for the identification of susceptible individuals and the development of effective pharmacotherapies. In humans, the level of response to alcohol is strongly predictive of subsequent alcohol abuse. Level of response is a combination of counteracting responses to alcohol, the level of sensitivity to the drug and the degree to which tolerance develops during the drug exposure, called acute functional tolerance. We use the simple and well-characterized nervous system of *Caenorhabditis elegans* to model the acute behavioral effects of ethanol to identify genetic and environmental factors that influence level of response to ethanol. Given the strong molecular conservation between the neurobiological machinery of worms and humans, cellular-level effects of ethanol are likely to be conserved. Increasingly, variation in long-chain polyunsaturated fatty acid levels has been implicated in complex neurobiological phenotypes in humans, and we recently found that fatty acid levels modify ethanol responses in worms. Here, we report that 1) eicosapentaenoic acid, an omega*-*3 polyunsaturated fatty acid, is required for the development of acute functional tolerance, 2) dietary supplementation of eicosapentaenoic acid is sufficient for acute tolerance, and 3) dietary eicosapentaenoic acid can alter the wild-type response to ethanol. These results suggest that genetic variation influencing long-chain polyunsaturated fatty acid levels may be important abuse liability loci, and that dietary polyunsaturated fatty acids may be an important environmental modulator of the behavioral response to ethanol.

## Introduction

Alcohol use disorders (AUD) are serious and widespread problems that affect approximately seventeen million Americans [1]. Genetics and environment both make significant contributions to the propensity to develop alcoholism [2], but the genes and specific environmental factors influencing abuse liability remain poorly understood. One problem with understanding the etiology of alcoholism is defining a quantitative phenotype that is tractable for study. The heritable phenotype of initial level of response (LR) to ethanol is a strong predictor of the subsequent development of alcohol use disorders [3]–[6], so factors influencing LR may also be central to the development of AUD, making them particularly attractive targets for research. Environmental risk factors alter alcohol abuse liability through interactions with genetic predispositions [7]. While previous studies of environmental contributors to alcoholism have considered a large variety of psychological and social factors that influence behavior, less attention has been paid to environmental factors that modify the physiological phenotype of LR. Here, we explore a possible role for diet in modulating LR using the nematode *Caenorhabditis elegans*.

*Caenorhabditis elegans* provides a simple system in which to study the genetic and molecular effects of ethanol intoxication and LR [8], [9]. Intoxication in *C. elegans* occurs at doses that cause intoxication in other organisms [10]. Genes that alter responses to ethanol in the worm also influence ethanol responses in rodents [8], [9], [11]–[14], suggesting that there are conserved mechanisms for ethanol responses between *C. elegans* and mammals. Initial LR is quantitative measure of the degree of intoxication at a particular alcohol concentration, e.g. alcohol-induced body sway in humans [6]. There are likely to be multiple physiological factors that influence LR, including neuronal homeostatic mechanisms acting in opposition to the drug’s effects. In both mammals and *C. elegans*, the measured LR is dependent on both the initial sensitivity to the effects of the drug and the degree to which tolerance to those effects develops during the alcohol exposure [8], [9], [15]–[17]. The within-session tolerance is called acute functional tolerance (AFT); it is observed in *C. elegans* and mammals, including humans. Different rates of development of AFT impact an individual’s level of response to alcohol. Previous work aimed at identifying the mechanisms of action for ethanol has shown that the effects of ethanol in *C. elegans* are largely in the nervous system and that initial sensitivity and AFT are dependent on proper neuronal expression of ethanol targets [8], [9], [16].

Recently, we observed that altering fatty acid levels in *C. elegans* modulates the development of AFT, and our results suggested that there were likely to be particular species of fatty acids that are important for AFT [16]. Here we have focused on the role of long-chain polyunsaturated fatty acids, (LC-PUFAs) in the behavioral responses to ethanol. LC-PUFAs are enriched in the brain and retina in mammals [18], [19], and mutations that alter fatty acid metabolism in worms have been shown affect neurotransmission by decreasing synaptic vesicle recycling and neurotransmitter release in *C. elegans* [20], [21]. Fatty acid metabolism is extremely well conserved between *C. elegans* and mammals, and mutations in the fatty acid metabolic pathway in worms have been characterized [22]–[24]. In this study, we assessed the role of LC-PUFAs on initial sensitivity and development of AFT in *C. elegans*.

## Results

### Long chain polyunsaturated fatty acids (LC-PUFAs) are required for the acute behavioral response to ethanol

**Figure 1:**
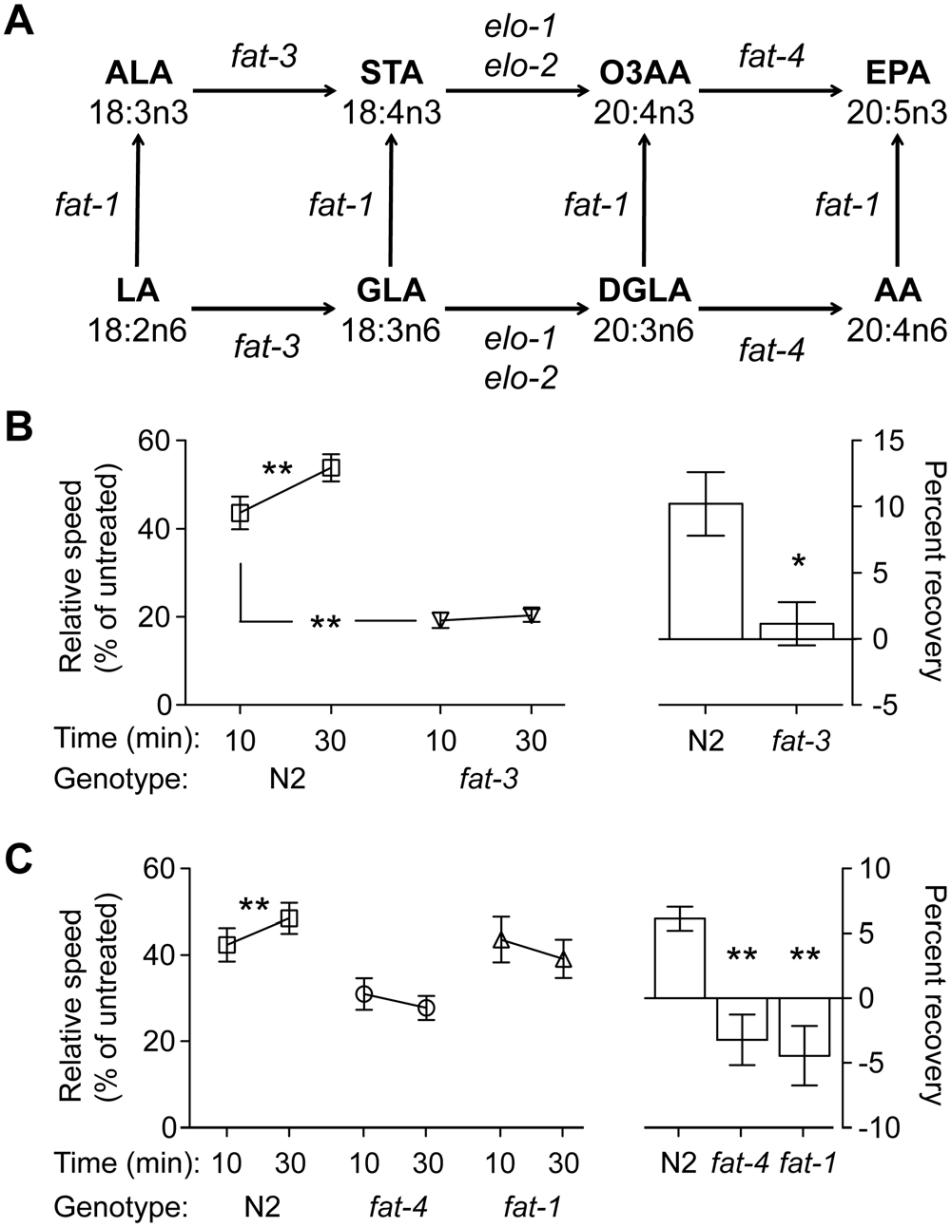
Long-chain polyunsaturated fatty acids are required for the development of acute functional tolerance to ethanol. (A) The metabolic pathway for LC-PUFAs in *C. elegans*. Genes encoding enzymes responsible for each step of the generation of each LC-PUFA are shown over the arrows. Mutations in genes encoding the enzymes FAT*-*3, FAT*-*4 and FAT*-*1 eliminate downstream LC-PUFAs [23], [26]–[28]. (B–C) N2 animals develop acute functional tolerance (AFT) to ethanol, whereas *fat-3(wa22), fat-4(wa14),* and *fat-1(wa9)* fail to develop AFT. Animals were treated with 0 or 400 mM ethanol, and locomotion was recorded at 10 and 30 minutes of exposure. Here and in subsequent figures, left graphs show relative speeds (treated/untreated speed). A difference at 10 minutes between N2 and the mutant indicates that the mutant has a change in initial sensitivity; a significant increase in speed from 10 to 30 minutes within a strain is defined as the development of AFT. Right graphs show percent of speed recovered between 10 and 30 minutes within a strain; this is the degree of AFT. *fat-3(wa22)* is more sensitive than wild type to the intoxicating effects of ethanol at 10 minutes in this set of experiments, although in subsequent experiments, this difference did not reach statistical significance. Error bars represent SEM. *p<0.05; **p<0.01. *n* = 6.

To determine if LC-PUFAs have a role in behavioral responses to ethanol, we exposed mutants lacking all or some LC-PUFAs to ethanol and assessed their locomotion behavior over a 30-minute exposure. In this acute ethanol exposure paradigm, we can observe both initial sensitivity (the degree to which animals are impaired by ethanol at 10 minutes) and the development of acute functional tolerance (AFT) to ethanol. AFT is a metabolism-independent compensation for the depressing effects of ethanol on locomotion [9]. Initial sensitivity and AFT are components of the acute level of response to ethanol [15], [17]. The development of AFT to the depressing effects of ethanol on locomotion speed in *C. elegans* is visible as an increase in the average speed after 30 minutes of ethanol exposure compared with the measured average speed at the initial 10-minute time point. The *fat-3* gene encodes a delta*-*6 fatty acid desaturase that is required for the generation of all LC-PUFAs in worms (Figure 1A) [23]. We found that *fat-3(wa22)* mutants were unable to develop AFT; unlike the wild-type animals, the measured speeds of the *fat-3* mutant animals were not different at the 10- and 30-minute time points (Figure 1B). This result suggests that one or more LC-PUFAs is necessary for normal acute behavioral responses to ethanol. Both arachidonic acid (AA) and eicosapentaenoic acid (EPA) are known to be involved in neuronal activity [20], [25], so we tested the requirement for these specific LC-PUFAs in the ethanol response using animals missing one or both of these fatty acids. The *fat-4* gene encodes a delta*-*5 fatty acid desaturase, and *fat-4(wa14)* mutants are unable to generate AA or EPA (Figure 1A) [26], [27]. *fat-4* mutant animals were unable to develop AFT to ethanol (Figure 1C), suggesting that AA and/or EPA is required for AFT. To distinguish between a requirement for AA or EPA, we tested animals carrying a mutation in the *fat-1* gene, which encodes an omega*-*3 fatty acyl desaturase that is required for the conversion of AA to EPA (Figure 1A). *fat-1(wa9)* mutants lack omega*-*3 arachidonic acid (O3AA) and EPA [26], [28]. *fat-1(wa9)* mutants were unable to develop AFT (Figure 1C), indicating that EPA and/or O3AA is required for normal AFT. Taken together, our data point to EPA as the LC-PUFA that is necessary for the normal development of AFT.

### EPA but not AA is necessary and sufficient for AFT

Dietary supplementation of PUFAs can rescue phenotypes associated with mutations in the biosynthetic enzymes for LC-PUFAs [20], [21], [26], [29]. *fat-1(wa9)*, *fat-4(wa14)*, and *fat-3(wa22)* mutants all display slow basal locomotion (Figure S1). Consistent with previous studies [20], [21], we found that raising *fat-3* mutant animals on medium containing AA over the course of their lifetime restored their basal speeds to wild-type. Supplementation of AA was also able to rescue the basal speeds of *fat-4* but not *fat-1* mutant animals, suggesting that the conversion of AA to EPA is necessary for normal locomotion speeds. Consistent with this hypothesis, supplementation of EPA was able to rescue the basal speeds of *fat-3*, *fat-4* and *fat-1* mutant animals (Figure S1).

**Figure 2:**
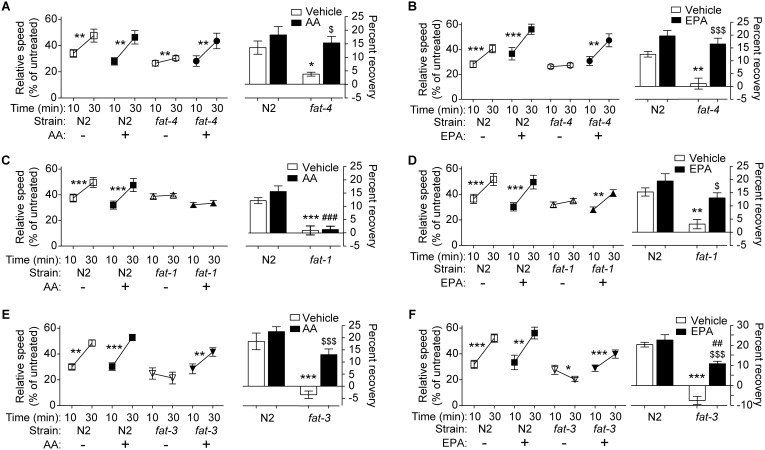
Dietary supplementation of EPA can rescue the AFT defect in mutants lacking LC-PUFAs. Animals were reared on NGM plates containing 0.1% NP*-*40 and 0 or 160 µM AA (A, C, E) or 0 or 160 µM EPA (B, D, F). (A, C, E) Supplementation with AA is able to rescue the AFT defect of *fat-4(wa14)* (A) and *fat-3(wa22)* (E), but not *fat-1(wa9)* (C) mutants, suggesting that the conversion of AA to EPA by FAT*-*1 is required for AFT. (B, D, F) Supplementation with EPA is able to rescue AFT in *fat-4(wa14)* (B), *fat-1(wa9)* (D) and *fat-3(wa22)* (F), indicating that EPA is sufficient to provide all LC-PUFA function that is required for AFT. Error bars represent SEM. *p<0.05; **p<0.01; ***p<0.001. *n* = 6 for AA supplementation and *n* = 5 for EPA supplementation.

We asked if dietary supplementation was able to restore wild-type responses to ethanol in these mutant backgrounds. *fat-4(wa14)* mutant animals fed AA are expected to accumulate both AA and EPA [23], [27], and we found that dietary supplementation of AA in *fat-4(wa14)* mutants restored their ability to develop robust AFT (Figure 2A). EPA supplementation of *fat-4(wa14)* should produce no AA [26]. EPA was also able to rescue the AFT defect of *fat-4(wa14)* (Figure 2B), suggesting that EPA and not AA is sufficient for AFT.

In order to further differentiate between the roles of AA and EPA in AFT, we supplemented *fat-1(wa9)* mutants with each LC-PUFA. *fat-1(wa9)* mutant animals lack EPA and O3AA [26]. As expected, EPA supplementation of *fat-1(wa9)* rescued AFT in this mutant (Figure 2D), suggesting that there is no requirement for O3AA in AFT. Supplementation of AA into *fat-1(wa9)* mutants, which cannot convert AA to EPA (Figure 1A) resulted in no significant development of AFT (Figure 2C), indicating that AA itself is insufficient to provide the LC-PUFA requirement in AFT.

To determine if EPA is the only LC-PUFA required for the development of AFT, we supplemented EPA into the diet of *fat-3(wa22)* worms, which lack all LC-PUFAs [23]. This supplementation has been shown to result in the accumulation of greater than wild-type levels of EPA while not affecting the levels of gamma-linolenic acid (GLA), dihomo-gamma-linolenic acid (DGLA), AA, and O3AA [26]. Supplementation of EPA in *fat-3(wa22)* worms was able to rescue the development of AFT (Figure 2E). Taken together, these results suggest that EPA is necessary and sufficient for the development of AFT.

### LC-PUFAs are required only in adults for AFT, but are required during development for normal basal locomotion

**Figure 3:**
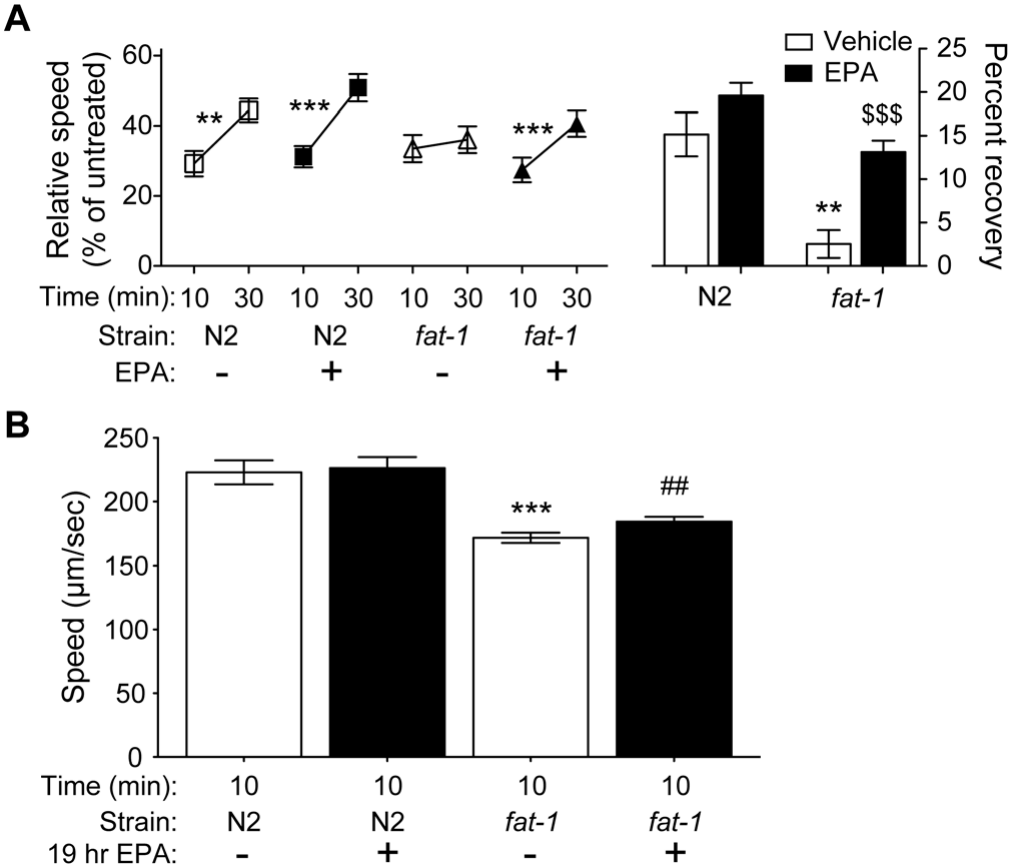
Nineteen hour treatment of *fat* *-* *1(wa9)* with EPA rescues AFT but not basal speed defects in *fat* *-* *1*. N2 and *fat-1(wa9)* mutant animals were grown to the L4 stage on NGM plates, then they were moved to NGM plates containing 0.1% NP*-*40 and supplemented with 0 or 160 µM EPA and allowed to develop into first day adults. After 19 hours of EPA supplementation, adult animals were tested in locomotion assays on 0 or 400 mM ethanol. (A) After 19 hours of EPA supplementation, *fat-1(wa9)* animals are able to develop AFT, whereas age-matched *fat-1(wa9)* animals not supplemented with EPA do not develop AFT. (B) 19 hours of EPA supplementation is not sufficient to rescue the slow basal speed of *fat-1(wa9)* mutant animals. Basal speed (0 mM ethanol) was measured at 10 and 30 minutes after the beginning of the locomotion assay. Error bars represent SEM. **p<0.01; ***p<0.001 within treatment; ##*p*<0.01 for comparison of supplemented *fat-1* to supplemented N2. *n* = 6.

LC-PUFAs are involved in neuronal development as well as in the function of mature neurons [20], [21], [25], [26], [29], [30]. To this point, our dietary supplementation studies all provided LC-PUFAs to animals throughout their development. In order to distinguish between developmental or acute requirements for EPA in AFT, we provided EPA to *fat-1* mutant animals at the last larval stage (L4), after most tissue development and differentiation has occurred. Consistent with a model in which EPA is required for adult neuronal function, we found that 19 hours of EPA supplementation (only during the L4 and early adult stages) was sufficient to rescue AFT in *fat-1(wa9)* (Figure 3A). This result strongly suggests that EPA is required in the mature nervous system for AFT, and that EPA is not required during development to make animals capable of developing AFT.

To our surprise, we found that this late supplementation of EPA was unable to rescue the slow basal speeds in *fat-1(wa9)* mutant animals (Figure 3B), suggesting that the basal speed defects in these animals are due to developmental requirements for EPA. Together, these data indicate that there are at least two separable requirements for EPA in normal adult behavior.

### The role of EPA in cholinergic signaling is distinct from its role in AFT

PUFAs are enriched in synaptic membranes [31] and their loss in a *fat-3* mutant results in a decrease of cholinergic neurotransmission, which can be rescued by AA supplementation [20], [21]. Modulation of neurotransmission activity is a potential mechanism for the development of AFT to ethanol. To assess if the function of LC-PUFAs in cholinergic neurotransmission overlaps with the role of EPA in the development of AFT, we tested the sufficiency of dietary supplementation of AA and EPA in rescuing the cholinergic defects of *fat-1* and *fat-4* mutants. The acetylcholinesterase inhibitor aldicarb inhibits the degradation of acetylcholine and results in tonic paralysis; the time necessary for paralysis is indicative of the amount of acetylcholine being released. Consistent with previous reports that observed aldicarb resistance in *fat-3* mutants [20], we found that *fat-1(wa9)* and *fat-4(wa14)* mutants are both resistant to aldicarb compared to wild-type animals (Figure S2A). Supplementation with EPA or AA was able to rescue aldicarb sensitivity in both mutants (Figure S2C–D) indicating that both AA and EPA are capable of restoring normal cholinergic signaling in animals deficient in LC-PUFAs. This is in contrast to our finding that AA fails to rescue the AFT defect in *fat-1(wa9)*, and indicates that the requirements for LC-PUFAs in AFT and cholinergic neurotransmission are not identical.

### Wild-type AFT can be modulated by dietary fatty acids

**Figure 4:**
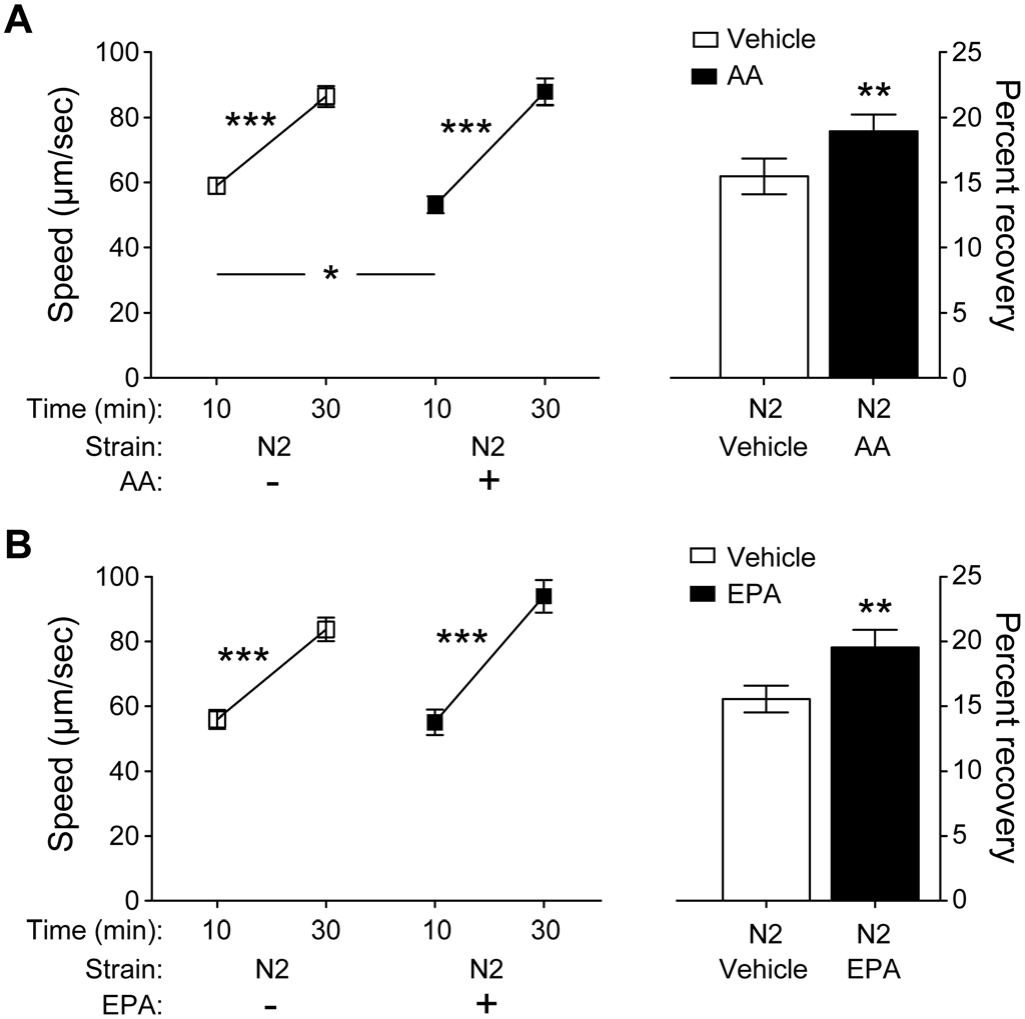
Dietary supplementation of AA or EPA can enhance the development of AFT in wild-type animals. All trials in which N2 was supplemented with AA or EPA are shown, *n* = 21 for AA, *n* = 18 for EPA. (A) N2 animals supplemented with AA demonstrate increased initial sensitivity to ethanol and enhanced development of AFT. (B) N2 animals supplemented with EPA demonstrate enhanced AFT. Error bars represent SEM. *p<0.05; **p<0.01; ***p<0.001.

Given the strength of the effect of altering EPA levels on AFT, dietary supplementation of EPA with wild-type animals that have normal endogenous levels of EPA may be expected to result in an enhancement of AFT. We noted that while supplementation of AA or EPA into wild-type N2 did not result in statistically significant changes in AFT in any set of the above experiments, in each case there was a strong trend toward dietary supplementation of LC-PUFAs causing an increase in the degree of AFT in wild-type animals. We suspected that increasing the number of trials would reveal a significant effect of dietary supplementation on wild-type responses to ethanol. When we compiled the wild-type response data from all supplementation experiments, we observed a significant increase in AFT when either EPA or AA was supplemented in N2 (Figure 4). Importantly, we observed no change in basal speeds in these animals with dietary supplementation (Table S1), suggesting that the effect of AA and EPA on AFT in wild-type animals is specific to the ethanol response mechanism. These results demonstrate that the intact molecular pathways involved in the development of AFT can be further modulated through dietary supplementation of LC-PUFAs.

## Discussion

The goal of this work was to determine if there is a role for LC-PUFAs in the acute response to alcohol. We measured the effects of acute ethanol exposure on the locomotion of *C. elegans*, and found that EPA modulates the development of AFT to ethanol. AFT is an example of neuronal plasticity, and the mechanisms underlying AFT represent a compensatory response to the environmental insult brought about by the actions of ethanol. This plasticity is independent of ethanol metabolism and most likely represents changes in neuronal function [8], [10]. The effects of fatty acids on AFT may therefore have relevance for other forms of homeostasis and plasticity.

We found that animals that are deficient in EPA are unable to develop AFT to ethanol. Dietary supplementation of EPA is sufficient to restore AFT to mutant animals that are unable to synthesize LC-PUFAs. AA is also able to modulate AFT, but only when it can be converted to EPA. The function of EPA in AFT appears to be independent of developmental roles of LC-PUFAs, as providing EPA to late larval stage and adult animals is sufficient for the generation of AFT. In contrast, LC-PUFAs are required developmentally for normal adult speeds in the absence of ethanol. LC-PUFA supplementation has been shown to influence cholinergic signaling possibly through interactions with synaptojanin [21]. Intriguingly, AA and EPA can both rescue cholinergic signaling independent of the conversion of AA to EPA. This suggests that the role for EPA in regulating AFT is not identical to its role in regulating cholinergic neurotransmission. Our finding that EPA alone is required for AFT differs from published work on other known roles of LC-PUFAs in worm behaviors. Kahn-Kirby *et al.* [26] demonstrated that either EPA or AA was able to modulate the functions of both the AWC and ASH sensory neurons. There is a clear requirement for LC-PUFAs in growth, basal locomotion and reproduction, because *fat-3* mutant animals have defects in these phenotypes. Both AA and EPA are able to rescue the gross phenotypic defects in *fat-3* mutant animals [29], although this did not explicitly test a requirement for AA because the functional *fat-1* in these animals means that AA supplementation would also provide EPA in these experiments. In addition to the requirement for EPA in AFT, there are other requirements for specific LC-PUFAs in worms. Recently, Vasquez *et al.* [25] demonstrated that AA is required for proper touch cell function in *C. elegans*. Together, these diverse findings demonstrate that different LC-PUFAs can have distinct roles in different neurobiological processes. Finally, we have shown that the manipulation of LC-PUFA levels by dietary supplementation in wild-type animals can alter the acute behavioral responses to ethanol. Together, these data highlight this environmental variable as a potential modifier of ethanol responses that are predictive of abuse liability.

One mechanism by which EPA may modulate AFT is through effects on membrane structure or function. Dietary omega*-*3 fatty acids, such as DHA and EPA, alter both lipid raft structure and the signaling of proteins known to reside in lipid rafts [32] and this may have functional consequences for interacting proteins that are involved in AFT. Lipids in the surrounding membrane modulate the functions of many proteins that are known to respond to ethanol. For example, we have previously identified the SLO*-*1 BK channel as an ethanol target in *C. elegans* [8], and mammalian BK channel activity is modulated by lipids [33]–[39]. We identified a genetic interaction between SLO*-*1 and the triacylglyceride lipase, *lips-7*, suggesting that the lipid milieu can modulate BK function *in vivo* in worms [16]. While modulation of SLO*-*1 function is not the only mechanism of AFT [16], if EPA modifies the function of it and/or other ethanol responsive proteins directly or indirectly though changing membrane characteristics, then the acute behavioral response to ethanol may be modified by dietary intake of PUFAs.

A second possibility is that LC-PUFAs are acting as signaling molecules or as precursors to signaling molecules that modulate ethanol responses. AA and EPA are metabolized into a variety of signaling molecules including eicosanoids and the resolvins. Interestingly, in *C. elegans*, the primary LC-PUFA precursor for the generation of eicosanoids is EPA, whereas in humans the primary precursor is AA [40]. While we have not yet determined if the requirement for EPA in AFT requires its metabolism to eicosanoids, there is increasing evidence for important roles for LC-PUFA derived eicosanoids in behavior in worms. EPA derived eicosanoids were found to regulate pharyngeal pumping [41], a neuromuscular behavior that is also affected by ethanol [8]. Furthermore, LC-PUFA derived eicosanoids have been found to be required for the behavioral response to reoxygenation after hypoxia [42]. Very recently, prostaglandin E2 has been demonstrated to regulate the function of the BK channel in mammals [43].

EPA could also be acting as a signaling molecule itself; Khan*-*Kirby *et al.* [26] demonstrated that exogenously applied EPA could acutely rescue a TRPV-dependent sensory response in worms, indicating that it could directly alter the function of TRPV channels. If EPA is acting as a signaling molecule in AFT, then its function differs from its signaling function with TRPV channels, because Kahn*-*Kirby *et al.* [26] were able to use either EPA or AA to modulate TRPV channels. Here we demonstrate that EPA and not AA is required for AFT.

In humans, most omega*-*3 PUFAs are derived from dietary sources, and diet can strongly influence the amount of omega*-*3 fatty acids in tissue reviewed in [44]. Millions of people routinely take fish oil supplements containing EPA, which can significantly increase plasma EPA levels [44]. There has been increasing interest in the relationship between neurobiological disorders and levels of omega*-*3 fatty acids. Several studies have demonstrated abnormal omega*-*3 fatty acid metabolism in depressed patients relative to controls [45], [46], and decreases in omega*-*3 fatty acids have been observed to correlate with deficiencies in dopaminergic signaling [47]. Major depressive disorder and alcohol abuse disorders (AUD) have been shown to be co-morbid [1], [48] and the dopamine pathway has been shown to respond profoundly to both acute and chronic ethanol [49]–[53]. Although there have been few studies on the relationship between omega*-*3 fatty acids and AUD, in one study, three weeks of dietary supplementation with EPA and DHA was found to significantly reduce stress and cortisol levels in abstinent alcoholics [54].

There are both genetic and environmental influences on the propensity to develop AUDs. The effect of fatty acids on ethanol responses may also be modulated by genetics. There is a significant genetic contribution to the acute level of response in humans [55]. Naturally occurring genetic polymorphisms in the fatty acid metabolism enzyme-encoding genes have been shown to detectably alter lipid profiles in humans [56]. Our work suggests that genetic variation in genes that modulate the physiological profile of LC-PUFAs could impact the acute response to alcohol, and therefore such genes represent potential risk loci.

Little is known about the environmental influences on the behavioral responses to alcohol. Our data indicate that by simple dietary supplementation with EPA, we can make measurable changes in acute ethanol tolerance in wild-type populations in *C. elegans*. In humans, acute ethanol tolerance is strongly correlated with abuse liability. Taken together, this and previous studies suggest that lipid metabolism and profile can influence responses to alcohol, and provide strong evidence for diet to be considered as a possible environmental risk factor in the liability for alcohol use disorders.

## Experimental Procedures

### Nematode husbandry

Unless otherwise noted, strains were maintained on nematode growth media (NGM) plates at 20°C. Before use, NGM plates were seeded with OP50 *E. coli* and a lawn of bacteria was allowed to grow overnight at room temperature. Strains used in this study were: Wild-type N2 (var Bristol), BX24 *fat-1(wa9)*, BX17 *fat-4(wa14)*, BX30 *fat-3(wa22)*.

### Locomotion tracking

Speed was analyzed as described previously [9] with minor changes: Assay plates (NGM) were dried for one hour at 37°C with the lids removed. Four copper rings were heated and melted into the surface of the agar. For ethanol treatment plates, ice-cold 100% ethanol was added to a final concentration in agar of 400 mM. Previously, we showed that a 10-minute 400 mM exogenous ethanol exposure produces a tissue concentration of ethanol of approximately 44–67 mM [10]. Plates were immediately sealed with Parafilm and the ethanol was left to absorb into the plate for two hours at room temperature. Age-matched first day adults were acclimated by moving them to unseeded plates with copper rings for thirty minutes. At thirty minutes ten worms of each strain were then moved from acclimation plates to the corresponding copper ring on the assay plate. Locomotion was recorded at 10 and 30 minutes of exposure. Movies were made on a Leica MZ6 stereo microscope with a 0.5x objective and 0.8x magnification using a Retiga 4000R camera (QImaging) and ImagePro Plus (6.2) (MediaCybernetics) software. Recordings were made at one frame per second and the speed of each worm was determined using ImagePro Plus software. The average speed for each group of 10 animals was calculated and treated as a single trial. All strains were tested a minimum of five times.

### Fatty acid supplementation

Fatty acid supplementation was performed as previously described [26]. Fatty acid salts (arachidonate and eicosapentaenoate; Nu-Chek Prep, Elysian, MN) were diluted to 20 mM in dH_2_O. NGM solutions were prepared with the addition of 0.1% NP*-*40 (Sigma), autoclaved, and maintained in a 60°C water bath. EPA or AA was added to a final concentration of 160 µM, control plates were supplemented with an equal volume of water. Plates were poured immediately and dried overnight at room temperature in a dark box. Plates were seeded with bacteria the following day and stored at 20°C for 48 hours before use.

The NP*-*40 vehicle itself causes a decrease in the overall speed of all animals that are exposed to it but does not affect animals in a genotype specific manner (Table S1). In each experiment using NP*-*40, the control animals were reared on plates containing NP*-*40 to account for this general slowing.

### Age synchronization

Egg laying: First day adult worms were allowed to lay eggs for two hours on seeded NGM or fatty acid supplemented plates. The adults were then removed and eggs were allowed to hatch and develop to first day adults before assaying.

### Assessment of ethanol response

A relative speed for each time point is calculated by dividing the speed of animals on ethanol (treated speed) by the speed of animals of the same genotype at the same time points on 0 mM ethanol (untreated speed). Strains that were compared were tested in parallel at the same time. We assess initial sensitivity by comparing the ten minute relative speeds between strains. A statistical difference in the speeds reflects a significant change in the initial sensitivity. We define a statistically significant increase in the relative speed at 30 minutes versus 10 minutes as the development of AFT. Degree of AFT is quantified as percent recovery of speed and is calculated by subtracting the 10-minute relative speed from the 30-minute relative speed.

### Statistics

Animals that were compared to each other were treated on the same plate at the same time under identical conditions. Data were converted to relative speeds to account for basal speed differences between strains (Table S1). Statistics were performed using Prism 5.0 (GraphPad). For comparisons of a single strain or treatment to a control, two-tail paired t-tests were performed. When multiple comparisons were necessary, as in the fatty acid supplementation assays, one-way ANOVA with a Dunnett’s post hoc test was used. Aldicarb assays were analyzed by two-way ANOVA with a Bonferroni post-hoc test.

### Aldicarb assay

Plates containing 1 mM aldicarb (Sigma) were prepared fresh for each set of paralysis assays as described previously [57]. Twenty adult animals were scored per experiment and three independent experiments were carried out [58]. Worms were scored at 30-minute intervals for three hours. They were counted as paralyzed if they were unable to respond to any of three pokes with a platinum wire on the head and tail.

## Supporting Information

Figure S1 EPA is required for normal speed of locomotion. Worms were reared on 0 or 160 µM AA (left graphs) or 0 or 160 µM EPA (right graphs) supplemented NGM plates containing 0.1% NP*-*40, and locomotion of first day adults was assessed. (A) *fat-3(wa22)* mutant animals lack all LC-PUFAs and have a slow locomotion phenotype. Dietary supplementation with AA (left) or EPA (right) can restore locomotion to wild-type speed. (B) *fat-4(wa14)* mutant animals lack AA and EPA, and dietary supplementation by either AA or EPA can restore wild-type locomotion speed. (C) *fat-1(wa9)* mutant animals lack EPA and cannot convert AA to EPA. Dietary supplementation with EPA but not AA is able to restore wild-type locomotion speeds to *fat-1* mutants, indicating that EPA is required for normal locomotion speed. Error bars represent SEM. **p<0.01; ***p<0.001 for comparison of unsupplemented mutant to unsupplemented N2; ###*p*<0.001 for comparison of supplemented mutant to supplemented N2; $$*p*<0.01, $$$*p*<0.001 for comparison of unsupplemented mutant to supplemented mutant. *n* = 6 for AA supplementation and *n* = 5 for EPA supplementation.
(PDF)

Figure S2 EPA and AA are required for acetylcholine signaling. Aldicarb is an acetylcholinesterase inhibitor that causes a progressive tonic paralysis that is dependent on the level of acetycholine (ACh) release. Worms were treated with 1 mM aldicarb and paralysis was assessed at 30-minute intervals. (A) *fat-1(wa9)* and *fat-4(wa14)* mutant animals are resistant to the paralyzing effects of aldicarb relative to N2, suggesting that they have decreased ACh neurotransmission. (B) EPA and AA supplementation do not alter aldicarb sensitivity in N2. (C–D) The aldicarb resistance of *fat-4(wa14)* mutant animals is rescued by dietary supplementation of either EPA or AA. (D) The aldicarb resistance of *fat-1(wa9)* is rescued by EPA or AA, demonstrating that AA alone is able to function to restore ACh signaling in animals lacking both AA and EPA. Filled symbols indicate time points that are statistically different (at least *p*<0.05) from N2 in (A), and from the non-supplemented animals in (B, C and D). (E) Dietary supplementation of AA or EPA are able to rescue the aldicarb resistance phenotypes of *fat-1* and *fat-4* animals at the 180 minute time point, while N2 is unaffected by the supplementation. *p<0.05; ***p<0.001 for comparison of unsupplemented mutant to supplemented mutant. Error bars represent SEM. *n* = 3.
(PDF)

Table S1 Raw locomotion data for all experiments. The raw locomotion data that is used to generate the relative speed measurements is presented. Each experiment includes an experimental group and its paired controls, which were reared in identical conditions and tested on the same plates at the same time. The treated and untreated animals for a given experiment were tested on the same day. There is some day-to-day variation in the absolute speeds of animals. In addition, the presence of NP*-*40 on the culture plates causes a general slowing of all genotypes of animals. In each case, the relative numbers that we present in the figures and text are derived from the experimental and their paired control animal data.
(PDF)
