# Supplementary figures and images for: The Omega-3 Fatty Acid Eicosapentaenoic Acid Is Required for Normal Alcohol Response Behaviors in C. elegans
Source: PLoS One. 2014 Aug 27;9(8):e105999. doi: 10.1371/journal.pone.0105999 (PMC4146551; doi:10.1371/journal.pone.0105999)

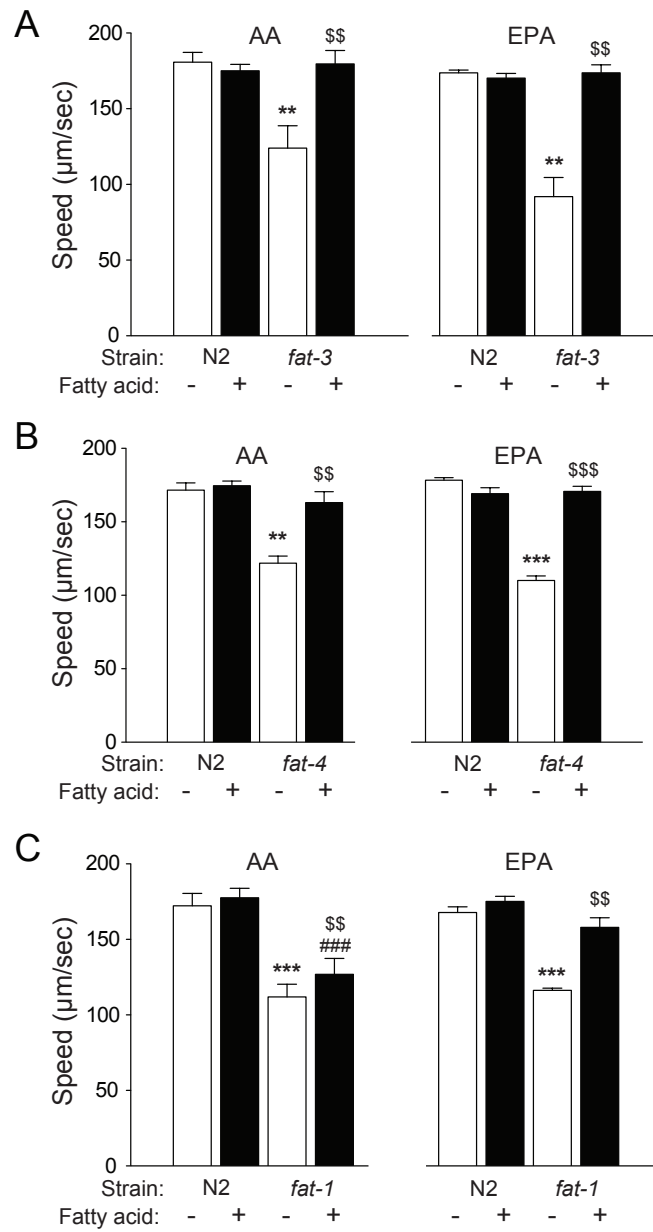

Raabe *et al.* Supplemental Figure S1

Supplement: Figure S1 — EPA is required for normal speed of locomotion. Worms were reared on 0 or 160 µM AA (left graphs) or 0 or 160 µM EPA (right graphs) supplemented NGM plates containing 0.1% NP-40, and locomotion of first day adults was assessed. (A) fat-3(wa22) mutant animals lack all LC-PUFAs and have a slow locomotion phenotype. Dietary supplementation with AA (left) or EPA (right) can restore locomotion to wild-type speed. (B) fat-4(wa14) mutant animals lack AA and EPA, and dietary supplementation by either AA or EPA can restore wild-type locomotion speed. (C) fat-1(wa9) mutant animals lack EPA and cannot convert AA to EPA. Dietary supplementation with EPA but not AA is able to restore wild-type locomotion speeds to fat-1 mutants, indicating that EPA is required for normal locomotion speed. Error bars represent SEM. **p<0.01; ***p<0.001 for comparison of unsupplemented mutant to unsupplemented N2; ###p<0.001 for comparison of supplemented mutant to supplemented N2; $$p<0.01, $$$p<0.001 for comparison of unsupplemented mutant to supplemented mutant. n = 6 for AA supplementation and n = 5 for EPA supplementation. (PDF) [file pone.0105999.s001.pdf]

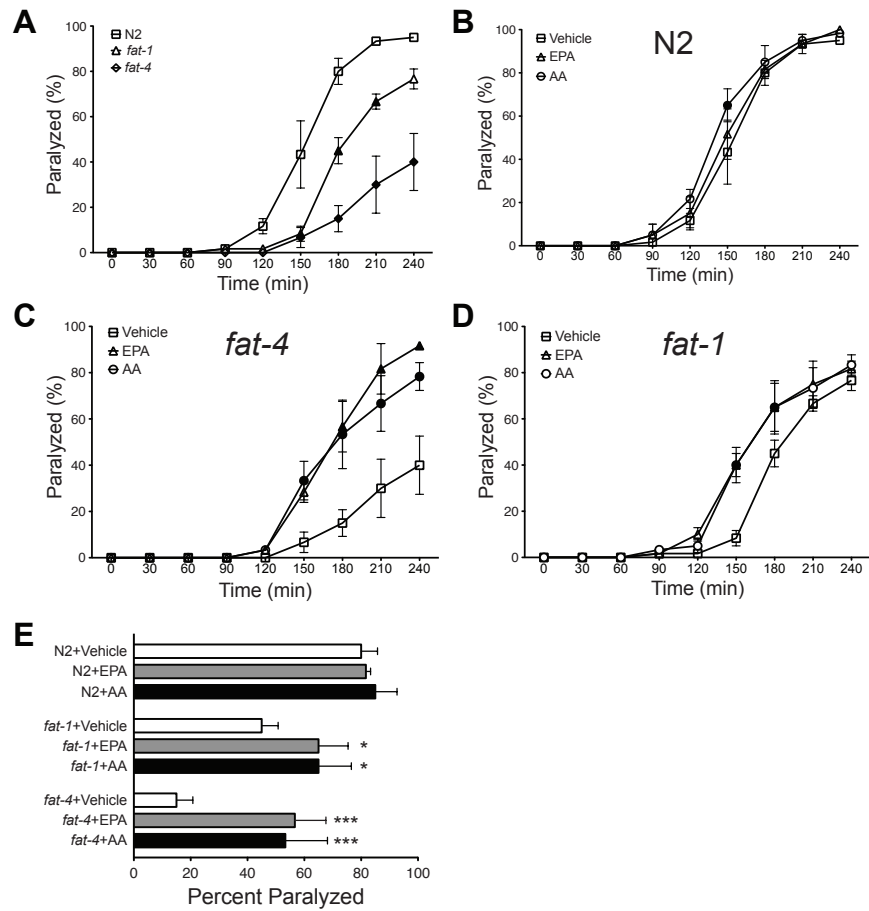

Raabe *et al.* Supplemental Figure S2

Supplement: Figure S2 — EPA and AA are required for acetylcholine signaling. Aldicarb is an acetylcholinesterase inhibitor that causes a progressive tonic paralysis that is dependent on the level of acetycholine (ACh) release. Worms were treated with 1 mM aldicarb and paralysis was assessed at 30-minute intervals. (A) fat-1(wa9) and fat-4(wa14) mutant animals are resistant to the paralyzing effects of aldicarb relative to N2, suggesting that they have decreased ACh neurotransmission. (B) EPA and AA supplementation do not alter aldicarb sensitivity in N2. (C–D) The aldicarb resistance of fat-4(wa14) mutant animals is rescued by dietary supplementation of either EPA or AA. (D) The aldicarb resistance of fat-1(wa9) is rescued by EPA or AA, demonstrating that AA alone is able to function to restore ACh signaling in animals lacking both AA and EPA. Filled symbols indicate time points that are statistically different (at least p<0.05) from N2 in (A), and from the non-supplemented animals in (B, C and D). (E) Dietary supplementation of AA or EPA are able to rescue the aldicarb resistance phenotypes of fat-1 and fat-4 animals at the 180 minute time point, while N2 is unaffected by the supplementation. *p<0.05; ***p<0.001 for comparison of unsupplemented mutant to supplemented mutant. Error bars represent SEM. n = 3. (PDF) [file pone.0105999.s002.pdf]
