# Supplementary material for: The Omega-3 Fatty Acid Eicosapentaenoic Acid Is Required for Normal Alcohol Response Behaviors in C. elegans
Source: PLoS One. 2014 Aug 27;9(8):e105999. doi: 10.1371/journal.pone.0105999 (PMC4146551; doi:10.1371/journal.pone.0105999)
Supplement: Table S1 — Raw locomotion data for all experiments. The raw locomotion data that is used to generate the relative speed measurements is presented. Each experiment includes an experimental group and its paired controls, which were reared in identical conditions and tested on the same plates at the same time. The treated and untreated animals for a given experiment were tested on the same day. There is some day-to-day variation in the absolute speeds of animals. In addition, the presence of NP-40 on the culture plates causes a general slowing of all genotypes of animals. In each case, the relative numbers that we present in the figures and text are derived from the experimental and their paired control animal data. (PDF) [file pone.0105999.s003.pdf]

Gene(allele)	Untreated Speeds (μm/sec)		Treated Speeds (μm/sec)	
	10'	30 '	10'	30'
fat-1(wa9)	169.7 ± 12.4	185.9 ± 5.9	71.1 ± 5.4	71.8 ± 7.0
Paired N2	242.3 ± 6.4	246.3 ± 5.6	102.4 ± 9.0	119.8 ± 10.0
fat-4(wa14)	148.0 ± 13.8	149.1 ± 5.1	45.1 ± 6.7	41.4 ± 4.7
Paired N2	242.3 ± 6.4	246.3 ± 5.6	102.4 ± 9.0	119.8 ± 10.0
fat-3(wa22)	133.3 ± 8.8	123.2 ± 6.2	25.2 ± 2.1	24.9 ± 2.0
Paired N2	240.7 ± 6.4	236.7 ± 4.4	104.3 ± 7.7	126.8 ± 5.9
fat-1(wa9) + NP-40	112.0 ± 8.4	114.1 ± 6.6	43.0 ± 2.8	45.1 ± 2.1
fat-1(wa9) + AA	126.9 ± 10.4	135.9 ± 11.8	41.0 ± 4.4	45.6 ± 5.3
Paired N2 + NP-40	173.1 ± 8.5	176.7 ± 5.5	64.6 ± 5.4	87.2 ± 6.0
Paired N2 + AA	177.6 ± 6.3	178.1 ± 4.4	56.4 ± 5.0	84.4 ± 8.5
fat-1(wa9) + NP-40	116.3 ± 1.4	120.4 ± 3.0	37.2 ± 2.2	42.3 ± 2.1
fat-1(wa9) + EPA	157.9 ± 6.4	162.2 ± 5.9	44.0 ± 3.6	66.1 ± 3.3
Paired N2 + NP-40	167.8 ± 3.8	174.3 ± 3.2	61.2 ± 6.4	90.0 ± 8.1
Paired N2 + EPA	175.2 ± 3.3	184.9 ± 4.2	52.6 ± 5.2	91.3 ± 9.0
fat-1(wa9) + NP-40 (19hr)	171.6 ± 4.1	167.4 ± 4.9	57.6 ± 6.8	60.2 ± 6.4
fat-1(wa9) + EPA (19hr)	184.2 ± 3.8	175.0 ± 4.0	50.9 ± 7.2	71.7 ± 8.4
Paired N2 + NP-40 (19hr)	222.9 ± 9.4	210.6 ± 8.2	65.2 ± 8.6	93.9 ± 8.4
Paired N2 + EPA (19hr)	226.3 ± 8.7	212.4 ± 9.5	78.0 ± 10.3	113.7 ± 12.9
fat-4(wa14) + NP-40	121.9 ± 4.9	117.2 ± 3.9	32.3 ± 3.0	35.7 ± 2.8
fat-4(wa14) + AA	163.1 ± 7.4	158.5 ± 6.6	45.0 ± 5.9	67.4 ± 7.5
Paired N2 + NP-40	172.7 ± 4.4	170.5 ± 4.0	59.0 ± 5.6	81.0 ± 8.0
Paired N2 + AA	174.5 ± 3.2	168.3 ± 4.6	48.7 ± 4.8	77.3 ± 8.2
fat-4(wa14) + NP-40	110.1 ± 3.0	112.1 ± 6.2	29.1 ± 2.2	30.6 ± 1.8
fat-4(wa14) + EPA	170.7 ± 3.5	172.9 ± 3.2	52.3 ± 5.5	81.8 ± 8.7
Paired N2 + NP-40	178.3 ± 1.7	177.1 ± 2.2	50.4 ± 5.0	72.2 ± 5.8
Paired N2 + EPA	169.2 ± 4.0	181.1 ± 4.4	61.3 ± 7.4	101.9 ± 7.6
fat-3(wa22) + NP-40	124.0 ± 14.7	118.7 ± 15.0	27.7 ± 2.0	22.2 ± 1.9
fat-3(wa22) + AA	179.6 ± 8.8	180.5 ± 5.3	50.9 ± 6.9	75.7 ± 7.6
Paired N2 + NP-40	180.7 ± 6.5	183.5 ± 7.1	54.2 ± 3.6	88.8 ±4.9
Paired N2 + AA	175.1 ± 4.2	182.7 ± 4.0	53.1 ± 5.5	96.5 ± 5.0
fat-3(wa22) + NP-40	92.0 ± 12.6	97.8 ± 12.0	23.8 ± 1.5	19.1 ± 1.3
fat-3(wa22) + EPA	173.7 ± 5.4	185.4 ± 3.1	49.8 ± 3.0	74.2 ± 6.9
Paired N2 + NP-40	173.7 ± 1.8	170.4 ± 4.2	55.4 ± 5.8	88.9 ± 5.6
Paired N2 + EPA	170.2 ± 3.1	175.8 ± 5.6	56.5 ± 9.1	99.6 ± 10.4
Compiled N2 + AA	173.6 ± 2.5	176.9 ± 2.5	53.2 ± 2.6	87.8 ± 4.1
Paired N2 + NP-40	175.0 ± 3.5	175.9 ± 2.9	59.0 ± 2.5	86.4 ± 3.1
Compiled N2 + EPA	172.1 ± 2.0	181.9 ± 2.4	55.1 ± 3.9	94.0 ± 5.0
Paired N2 + NP-40	173.1 ± 1.5	175.3 ± 1.8	56.0 ± 2.8	83.8 ± 3.6
